# Polymorphisms in the feline TNFA and CD209 genes are associated with the outcome of feline coronavirus infection

**DOI:** 10.1186/s13567-014-0123-6

**Published:** 2014-12-16

**Authors:** Ying-Ting Wang, Li-En Hsieh, Yu-Rou Dai, Ling-Ling Chueh

**Affiliations:** Graduate institute of Veterinary Medicine, School of Veterinary Medicine, National Taiwan University, Taipei, 10617 Taiwan; Department of Veterinary Medicine, School of Veterinary Medicine, National Taiwan University, Taipei, 10617 Taiwan

## Abstract

Feline infectious peritonitis (FIP), caused by feline coronavirus (FCoV) infection, is a highly lethal disease without effective therapy and prevention. With an immune-mediated disease entity, host genetic variant was suggested to influence the occurrence of FIP. This study aimed at evaluating cytokine-associated single nucleotide polymorphisms (SNPs), i.e., tumor necrosis factor alpha (TNF-α), receptor-associated SNPs, i.e., C-type lectin DC-SIGN (*CD209*), and the five FIP-associated SNPs identified from Birman cats of USA and Denmark origins and their associations with the outcome of FCoV infection in 71 FIP cats and 93 FCoV infected non-FIP cats in a genetically more diverse cat populations. A promoter variant, *fTNFA - 421 T*, was found to be a disease-resistance allele. One SNP was identified in the extracellular domain (ECD) of *fCD209* at position +1900, a G to A substitution, and the A allele was associated with FIP susceptibility. Three SNPs located in the introns of *fCD209*, at positions +2276, +2392, and +2713, were identified to be associated with the outcome of FCoV infection, with statistical relevance. In contrast, among the five Birman FIP cat-associated SNPs, no genotype or allele showed significant differences between our FIP and non-FIP groups. As disease resistance is multifactorial and several other host genes could involve in the development of FIP, the five genetic traits identified in this study should facilitate in the future breeding of the disease-resistant animal to reduce the occurrence of cats succumbing to FIP.

**Electronic supplementary material:**

The online version of this article (doi:10.1186/s13567-014-0123-6) contains supplementary material, which is available to authorized users.

## Introduction

Feline infectious peritonitis (FIP), a highly lethal disease with nearly 100% mortality among ill cats once clinical signs appear, is caused by feline coronavirus (FCoV) infection [1]. Despite the ubiquitous existence of FCoV around the world, the prevalence of FIP is less than 5% [2]. There is currently no therapy proven to be effective for the treatment of FIP, and once diagnosis is confirmed, euthanasia is generally inevitable. Although this disease has been described for over fifty years [3], studies attempting to develop vaccines with different approaches have all failed due to the immunopathogenic features of infection by this virus [4]. However, among many FCoV experimental inoculations studies, some cats survived challenge with the virulent strain of FCoV [2,5-10], whereas certain pedigreed cats were reported to be more likely to succumb to FIP than mixed bred cats [2,11,12]. All these findings indicate that genetic polymorphisms between cats might affect their susceptibility to FIP.

FIP is an immunopathological consequence of the abnormal production of various cytokines. Imbalanced Th1/Th2 immune responses with scarce or absent interferon-gamma (IFN-γ) is consistently found in FIP cases [4,8,10,13-15] and association of genetic polymorphisms in the IFN-γ gene with FIP occurrence has recently been identified [16]. In addition to IFN-γ, the upregulation of tumor necrosis factor-alpha (TNF-α) during the development of FIP has been reported to result in lymphopenia [17]. Feline dendritic cell (DC)-specific intercellular adhesion molecule-grabbing non-integrin (fDC-SIGN, encoded by *fCD209*), a key coreceptor during the infection of both type I and II FCoV [18], was found to affect binding and infection of type I FCoV. fDC-SIGN is also involved in the infection of type II FCoV, albeit not through the initial binding [19]. Despite the close relationship to FCoV infection, polymorphisms in the *fCD209* and feline TNF-α (*fTNFA*) genes and their association with FIP occurrence have never been investigated.

Recently, the surveillance of FIP-associated single nucleotide polymorphisms (SNPs) in Birman cats from USA and Denmark was conducted using a commercialized feline SNP array [20], and five SNPs were found to be significantly associated with FIP occurrence. However, it is unclear whether these disease-associated SNPs are Birman cat specific or can also be applied to other purebred or mixed breed cat populations.

To elucidate the genetic traits that contribute to FIP susceptibility, the *fTNFA* and *fCD209* genes were screened to identify disease-associated SNPs. The five SNPs identified from Birman cats proposed to be genetically associated with the occurrence of FIP were further evaluated in populations with more variable genetic backgrounds. Among all the polymorphisms analyzed, SNPs located in the *fTNFA* and *fCD209* genes were found to be associated with the outcome of FCoV infection, with statistical relevance.

## Materials and methods

### Animals and specimens

Samples were collected from 71 FIP cats and 93 FCoV-infected asymptomatic cats from 2005 to 2014 at the National Taiwan University Animal Hospital for an association analysis. This study required no specific ethical approval, as the analysis was performed retrospectively from samples of diseased animals routinely submitted to our diagnostic laboratory.

Seventy-one FIP cats, including 35.2% (25/71) purebred and 64.8% (45/71) mixed breed, were confirmed by necropsy. Pedigree cats including Scottish Fold (6/25), American Shorthair (4/25), Chinchilla (3/25), Exotic Shorthair (3/25), Siamese (2/25), European Shorthair (1/25) and Russian Blue (1/25). Most of the FIP cats (46/71) were less than one years old, the other 19 FIP cats were between the ages of one and three. The rest six FIP cats elder than three were from 3.5 to 10 years old. Also, FCoV detection by reverse transcription-nested polymerase chain reaction (RT-nPCR) [21] was confirmed in disease-associated tissue, including body effusions and/or internal organs with the typical lesions of FIP. Ninety-three asymptomatic healthy cats were included as a control group, including 75.3% (50/93) mixed breed and 24.7% (23/93) purebred, of an age of three years old or less and showing no FIP-related signs upon enrolment in this study. Pedigree cats including American Shorthair (8/23), Scottish Fold (7/23), Chinchilla (4/23), Persian (2/23), Abyssinian (1/23) and European Shorthair (1/23). All the asymptomatic cats were positive for FCoV detection in at least one sample collected, including whole blood, nasal/oral/conjunctival/rectal swabs, and feces. In addition, the detection of two feline retroviruses, i.e., feline leukemia virus and feline immunodeficiency virus, was performed [22,23]. FIP cats and FCoV infected non-FIP cats with a positive result for either of the feline retroviruses were excluded from the association study.

### Identification of SNPs in target sequences

Genomic DNA was isolated from buccal swabs or whole blood samples from each cat using a genomic DNA mini kit (Geneaid Biotech, New Taipei City, Taiwan). Partial *fTNFA* and *fCD209* sequences and five FIP-associated SNPs identified in Birman cats, as reported by Golovko et al., namely, *A1.196617776*, *A1.206840008*, *Un.59861682*, *A2.191286425*, and *E2.65509996*, were amplified [20] using the polymerase chain reaction (PCR). The primers and conditions are listed in Tables 1 and 2. Briefly, each reaction contained 1 μL of template DNA, 500 nM of each primer, 200 μM dNTP, 1.5 mM MgCl_2_, and 0.6 U Phusion DNA polymerase (Thermo Scientific, Waltham, USA) in a total volume of 30 μL with 1× Phusion HF buffer. The amplified products were subsequently sequenced using an auto-sequencer ABI 3730XL (Applied Biosystems, San Mateo, USA), and the obtained sequences were aligned by Geneious 4.8.5 (Biomatters, Auckland, New Zealand). The polymorphisms were further identified; the nucleotide positions of the SNPs are numerated from the translation start point (+1).

**Table 1:** **Primers used for SNP identification of** ***fTNFA*** **and** ***fCD209*** **gene**

**Target gene/region**	**Position** ^**a**^	**Orientation** ^**b**^	**Sequence (5′ - 3′)**	**T**_**A**_^**c**^	**Amplicon size**
*fTNFA*/5′-PRR^d^	−847 to −827	F	GAATTCCCAGGGTTGCTTTCA	65 °C	1018 bp
	+171 to +153	R	GCCGATCACTCCAAAGTGC		
*fCD209*/5′-PRR	−1057 to −1038	F	GAAGCGGGCTTCTTGTTGAC	65 °C	1076 bp
	+19 to +1	R	GCTCCTTGGGGTCACACAT		
*fCD209*/ECD^e^	+1818 to +1838	F	CCAAGATCTGATGCATCTGCT	67 °C	1350 bp
	+3168 to +3149	R	ATGAGCTCGTTGCCTGATCT		

^a^The nucleotide positions are numerated from the translation start point (+1).

^b^F: forward; R: reverse.

^c^Annealing temperature.

^d^5′-proximal regulatory region.

^e^Extracellular domain.

**Table 2:** **Primers used for SNP identification of five suspected FIP-associated SNPs in Birman cats**

**SNP name**	**Chr-SNP position** ^**a**^	**Orientation** ^**b**^	**Sequence (5′ - 3′)**	**T**_**A**_^**c**^	**Amplicon size**
*A1.196617776*	A1-154265118	F	GGCAGTCAGAGAATGAGACAC	61 °C	337 bp
		R	TTGCCAGTTCTGCAGATTG		
*A1.206840008*	A1-164728174	F	AGGTGAAGTGTTGTGTGCAT	61 °C	388 bp
		R	ATGTTCTGCTAGATGAGCCG		
*Un.59861682*	A1-155715831	F	CTCATCCCAGTTGATCACAC	61 °C	230 bp
		R	TTCCTCCTGGAAAACCCT		
*A2.191286425*	A2-126618108	F	AGCGTATCAAGTGCCTGC	61 °C	299 bp
		R	CCTTCCTGTTTAGGTGCTTG		
*E2.65509996*	E2-54165589	F	CGCTTCAGTTTCCTTTCCAG	61 °C	417 bp
		R	TCTGAGCCTTGGTCTTCTG		

^a^Chr: chromosome.

^b^F: forward; R: reverse.

^c^Annealing temperature.

### Association analysis

The association between the targeted SNPs and the occurrence of FIP was analyzed using Fisher’s exact test, and a *P* value < 0.05 was considered to be a significant association.

## Results

### Polymorphism at *fTNFA - 421* was found to be significantly associated with resistance to FIP

**Figure 1:**
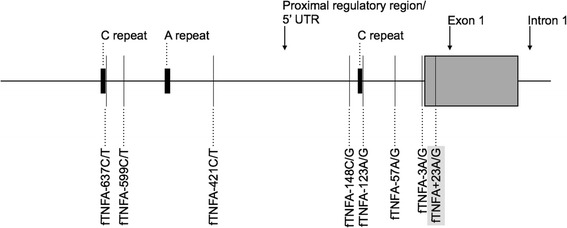
**A schematic of the 5′ terminal of the** ***fTNFA*** **gene analyzed in this study.** A partial *fTNFA* sequence of 1018 bp was sequenced in this study, including the PRR, the 5′-UTR, and part of exon 1. All the SNPs and the corresponding positions are indicated with lines. Gray box: exon 1. Black boxes: repeat regions. SNPs located in the exon were shaded.

Because the overproduction of TNF-α is widely reported in FIP animals and is considered to contribute to the pathogenesis of FIP, we first screened the polymorphisms at the 5′ terminus of the *fTNFA* gene, including the proximal regulatory region (PRR), the 5′-untranslated region (UTR), and part of exon 1, in 71 FIP and 93 control cats. Eight SNPs and three repeat regions were identified in the analyzed 1018 bp (Figure 1). One SNP located in exon 1 at position +23 results in the substitution of CGG to CAG, causing an amino acid change from Arg to Gly (R8G); the remaining SNPs were found in the PRR. The mean allele frequencies of the minor alleles ranged from 4.3% to 39.9%. To examine the association between the identified SNPs in *fTNFA* and the outcome of FCoV infection, the frequency of each genotype and allele was analyzed (Additional file 1). Only one allele (T allele) at position −421 appeared to be significantly associated with resistance to FIP (*P* = 0.009, OR = 3.925), whereas the others showed no significance to the disease (Table 3 and Additional file 1).

**Table 3:** **FIP-associated genetic polymorphisms identified in the** ***fIFNG*** **,** ***fTNFA*** **, and** ***fCD209*** **gene and their associations with FIP**

**Target gene/position**	**Susceptible allele**	**Resistant allele**	***P*** **value**	**OR** ^**a**^	**Reference**
*fIFNG +428*	C	T	0.03	3.4 (1.1-10.3)	[16]
*fTNFA - 421*	C	T	0.009	3.9 (1.3-11.8)	This study
*fCD209 + 1900*	A	G	0.014	3.7 (1.3-10.5)	This study
*fCD209 + 2276*	C	T	0.038	NA^b^	This study
*fCD209 + 2392*	G	A	0.016	2.6 (1.2-5.5)	This study
*fCD209 + 2713*	T	C	0.039	1.75 (1.1-2.9)	This study

^a^Odds Ratio.

^b^Not available.

### Polymorphisms in the extracellular domain and introns 6 and 7 of *fCD209* were found to be significantly associated with the disease outcome

**Figure 2:**
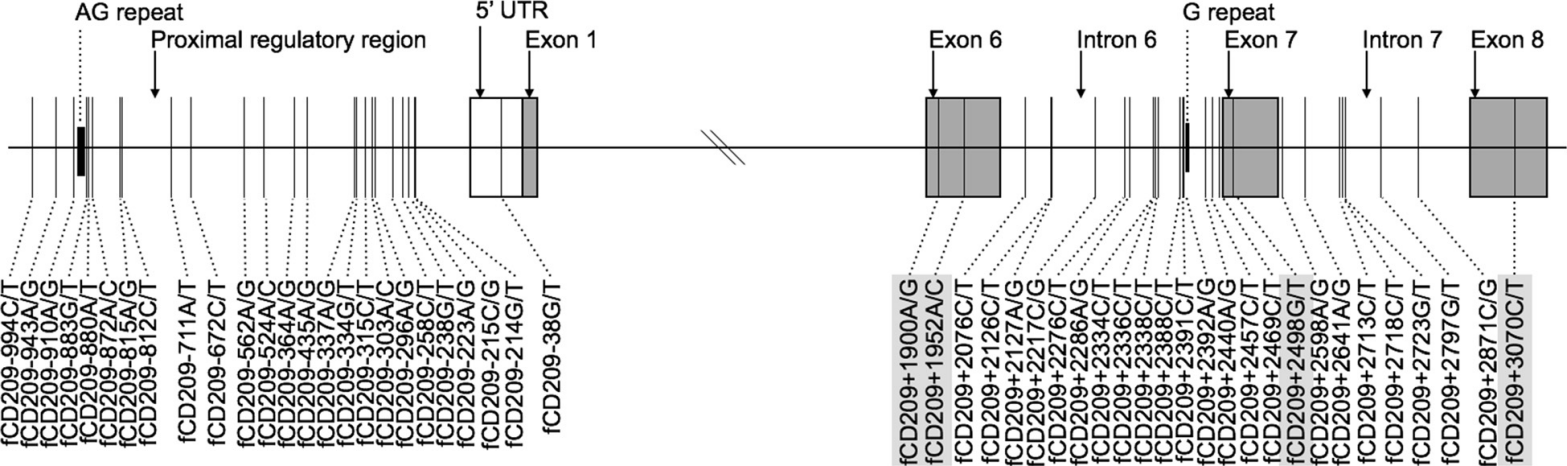
**A schematic of the partial** ***fCD209*** **gene analyzed in this study.** The PRR, the 5′-UTR, and the ECD of *fCD209* were sequenced in this study. All the SNPs and the corresponding positions were indicated with lines. Gray boxes: exons. Black boxes: repeat regions. White box: 5′ UTR. SNPs located in the exon were shaded.

*fCD209* is an important co-receptor for both type I and II FCoV infection. To demonstrate an association between polymorphisms and FIP, we sequenced polymorphisms of the PRR, 5′-UTR, and extracellular domain (ECD) of *fCD209* in 71 FIP and 93 control cats. Twenty-four SNPs and one AG repeat region were identified in the PRR, and one SNP was found in the 5′-UTR (Figure 2). Furthermore, polymorphism screening of ECD, revealed 25 SNPs and a G repeat in the 1350-bp region analyzed (Figure 2). Four SNPs were located in exons, including two SNPs in exon 6 at positions +1900 (TGG > TAG, W128*) and +1952 (AAC > AAA, N145K), one in exon 7 at position +2498 (ACG > ACT, T178T), and one in exon 8 at position +3070 (TTC > TCC, F241S). The remaining 21 SNPs were located in introns 6 and 7 (Figure 2). The mean allele frequencies of the minor alleles ranged from 0.6% to 47.6%.

To further demonstrate the association between the identified SNPs in *fCD209* and the outcome of FCoV infection, the frequency of each genotype and allele was analyzed. Among the 24 SNPs analyzed in the PRR and the 5′-UTR of the *fCD209* gene, no genotype or allele showed a significant association with the outcome of the infection (Additional file 2). In contrast, four SNPs, +1900 in the ECD of exon 6, +2276 and +2392 in intron 6, and +2713 in intron 7, identified in *fCD209* were found to be significantly associated with FIP. *fCD209 + 1900*, located in the ECD, which is characterized by a G-to-A substitution that leads to a premature stop codon (TGG > TAG, W128*), had a significantly increased frequency in FIP animals (18.31%) compared with the control animals (5.38%) (*P* = 0.011, OR = 3.95), and the A allele was shown to be significantly associated with susceptibility to FIP (*P* = 0.014, OR = 3.65) (Table 3 and Additional file 2). Furthermore, three SNPs, +2276, +2392, and +2713, located in introns 6 and 7 were found to be associated with disease susceptibility. A higher frequency of the T allele at position +2276 in the control cats was significantly associated with resistance to FIP (*P* = 0.038). Similarly, the A allele at position +2392, with a higher frequency in FCoV-infected asymptomatic cats, showed a significant association with disease resistance (*P* = 0.016, OR = 2.57). Moreover, the T allele at position +2713 was identified with a higher frequency in FIP cats, showing a significant association with disease susceptibility (*P* = 0.039, OR = 1.75) (Table 3 and Additional file 2).

### Evaluation of the association between the FIP-associated SNPs reported in Birman cats and disease susceptibility in a cat population with higher genetic variability

Five SNPs were reported to be associated with the occurrence of FIP in Birman cats in a recent study using genome-wide association analysis [20]. Due to the lack of information concerning the nucleotide sequence of the SNPs, the sequences were identified. The nucleotide sequences of *A1.196617776*, *A1.206840008*, *Un.59861682*, *A2.191286425*, and *E2.65509996* consisted of C/A, G/A, G/A, C/T, and C/T, respectively. To test for an association of these SNPs with the occurrence of FIP also applies to other breeds of cats, the frequency of each genotype and allele of the targeted SNPs was analyzed in all the FIP and control cats enrolled in this study, i.e., 29.3% purebred and 70.7% mixed breed. Among the five SNPs analyzed, neither the genotype nor allele percentage showed a significant association with the outcome of the disease (Additional file 3).

### Association study

Among all the FIP animals analyzed in this study, 64 (64/71, 90.1%) cats were found to be effusive form (wet), and the rest seven of them (7/71, 9.9%) were non-effusive (dry). The association between the identified FIP-related SNPs (*fTNFA* - *421*, *fCD209* + *1900*, + *2276*, + *2392* and +*2713*) and the form of FIP were analyzed. None of the SNPs was found to be associated with the biotype of FIP.

### The number of the FIP-associated SNPs harbored is correlated to the disease outcome

This study attempted to distinguish multiple genetic traits associated with FIP susceptibility. The FIP-associated genetic polymorphisms identified in the *fIFNG* [16], *fTNFA*, and *fCD209* genes are summarized in Table 3. Moreover, the association between the number of FIP-associated SNPs harbored, including resistant or susceptible genotype/alleles identified at *fIFNG +428*, *fTNFA - 421*, and *fCD209 + 1900*, *+ 2276*, *+ 2392*, and *+2713*, and the occurrence of FIP, was analyzed in this study (Table 4). The number of FIP-resistant SNPs carried was found to be associated with the protection of cats from FIP (*P* = 0.002), and the odds ratios of FIP and non-FIP cats carrying one or more (≥ 2) resistance SNPs were 3.06 and 6.01, respectively; this result indicates that cats carrying more resistant SNPs appear to have a lower chance of developing FIP. However, the FIP cats were identified with a higher frequency as carrying more than one FIP-susceptible SNP (50.7%) than the control cats (29.0%), showing a significant association with disease susceptibility (*P* = 0.0059, OR = 2.51) (Table 4).

**Table 4:** **Association of the number of the disease-associated SNPs harbored, including** ***fIFNG +428*** **,** ***fTNFA - 421*** **, and** ***fCD209 + 1900*** **,** ***+ 2276*** **,** ***+ 2392*** **, and** ***+2713*** **, and the outcome of FIP**

**Genotypes/alleles harbored**	**FIP number (%)**	**Non-FIP number (%)**	***P*** **value**	**OR (95% CI)**
Resistant SNPs				
= 0	59 (83.1%)	54 (58.1%)	0.002	Reference
= 1	10 (14.1%)	28 (30.1%)		3.06 (1.36 - 6.88)
≥ 2	2 (3%)	11 (11.8%)		6.01 (1.27 - 28.35)
Susceptible SNPs				
= 0	35 (49.3%)	66 (71.0%)	0.0059	Reference
≥ 1	36 (50.7%)	27 (29.0%)		2.51 (1.32 - 4.80)

## Discussion

FIP is an important infectious disease in cats, with nearly 100% mortality. However, an understanding of the host determinants in the occurrence of FIP has been limited to date. An imbalance between cellular and humoral immunity - with excess antibodies contributing to disease progression [4,24] and a significant decrease in IFN-γ production [4,8,10,13-15] - has been consistently observed in FIP animals. We recently identified the first host gene – *fIFNG* – showing an association between host genetic polymorphisms and FIP [16]. A T allele at *fIFNG +428* was identified as a resistant allele, and the heterozygous genotypes (*CT*) at positions +401 and +408 were identified as associated with susceptibility to type I FCoV-induced FIP. In this study, five additional SNPs from *fTNFA* and *fCD209* were identified.

During the development of FIP, the upregulation of TNF-α, an important pro-inflammatory cytokine, has been consistently found [4,17,24,25]. The overproduction of TNF-α induces apoptosis in CD8+ T cells and is associated with the upregulation of a type II FCoV receptor, i.e., feline aminopeptidase N, which accelerates macrophage infection by the virus [26]. Moreover, TNF-α together with granulocyte monocyte-colony stimulating factor (GM-CSF), G-CSF, and other neutrophil survival factors are suggested to prolong the survival of neutrophils, activate monocytes/macrophages, and contribute to the formation of the pyogranulomatous lesions of FIP [25]. In several human diseases, the most commonly identified genetic polymorphisms are located at position −238 and −308 of the promoter region of *TNFA*, suggesting an effect on the binding of transcription factors [27], with susceptibility for the development of several viral diseases, including severe acute respiratory syndrome (SARS) [28], dengue hemorrhagic fever (DHF) [29], and hepatitis B virus (HBV) infection [30]. Compared to the human gene (*TNFA - 308*), SNP located in a slightly upstream region of the feline gene (*fTNFA - 421*) was found to be significantly associated with the occurrence of FIP. The variant *fTNFA - 421 T* allele was significantly associated with resistance to FIP. Through DNA transcriptional factor binding site prediction, we found that an *fTNFA - 421* C to T mutation might affect the binding of some transcription factors, such as myeloid zinc finger 1 (MZF1) [31], a transcriptional regulator [32]. Through promoter binding, MZF1 was identified to function as a transcription activator of hematopoietic cells in vitro [32]. Because macrophages are hematopoietic cells, the loss of the binding site for such transcription factors might decrease TNF-α production in macrophages, which might prevent the immunopathogenesis and result in a resistant phenotype.

DC-SIGN, which recognizes high-mannose oligosaccharides as its ligand, is a co-receptor augmenting many viral infections, including human immunodeficiency virus [33], dengue virus [34], HBV [35], and SARS-coronavirus [36]. In addition, feline DC-SIGN also serves as a co-receptor and is involved in infection by FCoV [19]. A human SNP in the promoter region of *CD209* (−*336 A/G*) was identified as related to disease prognosis [36]. A variant of *CD209 - 336* was reported to affect the binding of SP1-like transcription factor and might modulate transcriptional activities [34], indicating that *CD209 - 336* plays a crucial role in disease pathogenesis. However, in our study, none of the SNPs in the promoter region showed an association with the outcome of FCoV infection. The identified FIP-associated SNPs, +1900 G/A, +2276 C/T, +2392 G/A, and +2713 C/T, were all located at the 3′ end of *fCD209*. The polymorphism identified at *fCD209 + 1900* is located in the lectin binding domain of the ECD; a G to A substitution leads to a change from a tryptophan at amino acid 128 to a stop codon. This mutation might lead to an abortive mRNA [37] or a truncated protein if the mRNA is successfully translated. DC-SIGN serves as a pattern recognition receptor that interact with numerous pathogens, including FCoV, and mediates the clustering of DC with naive T cells [38]. Additionally, with a type II transmembrane domain, the truncated fDC-SIGN identified in this study (+1900) might still be expressed on the cell surface. However, it remains to be elucidated how the truncated protein, bearing only one half of the authentic protein, affect the normal function of DC-SIGN, i.e., pathogen recognition or T cell activation. The other three FIP-associated SNPs were located in introns 6 and 7. Although these polymorphisms are located in introns, which apparently would not affect the protein, a regulatory effect cannot be excluded. In humans, the SNP at *IFNG +874*, located in intron 1, was found to alter the binding activity of nuclear factor kappa-light-chain-enhancer of activated B cells and influenced the production of IFN-γ [39]. In swine, an SNP (G3072A) in intron 3 of insulin-like growth factor 2 affected the binding of muscle growth regulator and was associated with the muscle content [40].

Several pedigreed cats, including Abyssinians, Himalayans, Birmans, Bengals, Ragdolls, and Rexes, were reported to have a higher incidence of FIP than other breed cats [2]. Recently, a genome-wide association study of Danish and American Birman cats populations identified five SNPs involved in FIP susceptibility [20]. However, none of these SNPs showed a similar correlation in the present study, concordant with the postulation by the authors that these associations might only be relevant to Birman breed or other breeds with similar genetic traits [24].

In addition to the three SNPs in *fIFNG*, we identified in this study five more SNPs from two genes - one in *fTNFA* and four in *fCD209* - that are associated with the occurrence of FIP. As the susceptibility or resistance to viral infections is a complex phenotype regulated by multiple interacting genes and gene networks, genes related to innate and adaptive immunity and other host genes remain to be pursued. The combination of all the FIP susceptibility genotypes into a single typing diagnosis assay should facilitate the screening of FIP-resistant cats in breeding and eventually decrease the loss of cats to this incurable disease.

## Additional files

Additional file 1:
**Frequencies of the** ***fTNFA*** **genotypes and alleles and associations with the outcome of FCoV infection.** The promoter variant, *fTNFA - 421 T*, was found to be a disease-resistance genotype. Additional file 2:
**Frequencies of the** ***fCD209*** **genotypes and alleles and associations with the outcome of FCoV infection.** Polymorphisms of *fCD209,* including *fCD209* + *1900*, + *2276*, + *2392* and +*2713*, were found to be significantly associated with the disease outcome. Additional file 3:
**Frequencies of the genotypes and alleles of the proposed FIP-associated SNPs in Birman cats and associations with FIP.** Neither the percentage of genotypes nor alleles was found to be associated with the outcome of FCoV infection in disease and non-disease group.
