# Supplementary material for: Polymorphisms in the feline TNFA and CD209 genes are associated with the outcome of feline coronavirus infection
Source: Vet Res. 2014 Dec 16;45(1):123. doi: 10.1186/s13567-014-0123-6 (PMC4267428; doi:10.1186/s13567-014-0123-6)
Supplement: Additional file 1: — Frequencies of the fTNFA genotypes and alleles and associations with the outcome of FCoV infection. The promoter variant, fTNFA - 421 T, was found to be a disease-resistance genotype. [file 13567_2014_123_MOESM1_ESM.doc]

**Additional file 1 Frequencies of the *fTNFA* genotypes and alleles and associations with the outcome of FCoV infection.**

| SNP | FIP number (%) | Control number (%) | OR (95% CI)^a^ | *P* value |
| --- | --- | --- | --- | --- |
| *fTNFA + 23* |  |  |  |  |
| *GG* | 37 (52.1) | 61 (65.6) |  | 0.218 |
| *GA* | 23 (32.4) | 22 (23.7) |  |  |
| *AA* | 11 (15.5) | 10 (10.8) |  |  |
| *G* allele | 97 (68.3) | 144 (77.4) |  | 0.077 |
| *A* allele | 45 (31.7) | 42 (22.6) |  |  |
| *fTNFA - 3* |  |  |  |  |
| *GG* | 69 (97.2) | 84 (90.3) |  | 0.208 |
| *GA* | 1 (1.41) | 7 (7.5) |  |  |
| *AA* | 1 (1.4) | 2 (2.2) |  |  |
| *G* allele | 139 (97.9) | 175 (94.9) |  | 0.105 |
| *A* allele | 3 (2.1) | 11 (5.9) |  |  |
| *fTNFA - 57* |  |  |  |  |
| *GG* | 40 (56.3) | 47 (50.5) |  | 0.355 |
| *GA* | 20 (28.2) | 23 (24.7) |  |  |
| *AA* | 11 (15.5) | 23 (24.7) |  |  |
| *G* allele | 100 (70.4) | 117 (62.9) |  | 0.160 |
| *A* allele | 42 (29.6) | 69 (37.1) |  |  |
| *fTNFA - 123* |  |  |  |  |
| *GG* | 64 (90.1) | 83 (89.3) |  | 0.561 |
| *GA* | 6 (8.5) | 6 (6.5) |  |  |
| *AA* | 1 (1.4) | 4 (4.3) |  |  |
| *G* allele | 134 (94.4) | 172 (92.5) |  | 0.657 |
| *A* allele | 8 (5.6) | 14 (7.5) |  |  |
| *fTNFA - 148* |  |  |  |  |
| *GG* | 35 (49.3) | 42 (45.2) |  | 0.083 |
| *GC* | 24 (33.8) | 22 (23.7) |  |  |
| *CC* | 12 (16.9) | 29 (31.2) |  |  |
| *G* allele | 94 (66.2) | 106 (57.0) |  | 0.110 |
| *C* allele | 48 (33.8) | 80 (43.0) |  |  |
| *fTNFA - 421* |  |  |  |  |
| *CC* | 68 (95.8) | 82 (88.2) |  | 0.137 |
| *CT* | 2 (2.8) | 3 (3.2) |  |  |
| *TT* | 1 (1.4) | 8 (8.6) |  |  |
| *C* allele | 138 (97.2) | 167 (89.8) | 3.925 (1.3 - 11.8) | 0.009 |
| *T* allele | 4 (2.8) | 19 (10.2) |  |  |
| *fTNFA - 599* |  |  |  |  |
| *TT* | 39 (54.9) | 41 (44.1) |  | 0.395 |
| *TC* | 14 (19.7) | 23 (19.7) |  |  |
| *CC* | 18 (25.4) | 29 (31.2) |  |  |
| *T* allele | 92 (64.8) | 105 (56.5) |  | 0.140 |
| *C* allele | 50 (35.2) | 81 (43.6) |  |  |
| *fTNFA - 637* |  |  |  |  |
| *CC* | 41 (57.8) | 49 (52.7) |  | 0.498 |
| *CT* | 5 (7.0) | 12 (12.9) |  |  |
| *TT* | 25 (35.2) | 32 (34.4) |  |  |
| *C* allele | 87 (61.3) | 110 (59.1) |  | 0.734 |
| *T* allele | 55 (38.7) | 76 (40.9) |  |  |

^a^ Odds Ratio.
