# Supplementary material for: Polymorphisms in the feline TNFA and CD209 genes are associated with the outcome of feline coronavirus infection
Source: Vet Res. 2014 Dec 16;45(1):123. doi: 10.1186/s13567-014-0123-6 (PMC4267428; doi:10.1186/s13567-014-0123-6)
Supplement: Additional file 2: — Frequencies of the fCD209 genotypes and alleles and associations with the outcome of FCoV infection. Polymorphisms of fCD209, including fCD209 + 1900, + 2276, + 2392 and +2713, were found to be significantly associated with the disease outcome. [file 13567_2014_123_MOESM2_ESM.docx]

**Additional file 2 Frequencies of the *fCD209* genotypes and alleles and associations with the outcome of FCoV infection.**

| SNP | FIP  number (%) | Non FIP number (%) | OR (95% CI)^a^ | *P* value |
| --- | --- | --- | --- | --- |
| *fCD209 - 38* |  |  |  |  |
| *GG* | 67 (94.4) | 85 (91.4) |  | 0.556 |
| *GT* | 4 (5.6) | 8 (8.6) |  |  |
| *TT* | 0 (0.0) | 0 (0.0) |  |  |
| *G* allele | 138 (97.2) | 178 (95.7) |  | 0.563 |
| *T* allele | 4 (2.8) | 8 (4.3) |  |  |
| *fCD209 - 214* |  |  |  |  |
| *TT* | 69 (97.2) | 90 (96.8) |  | 1.000 |
| *TG* | 2 (2.8) | 3 (3.2) |  |  |
| *GG* | 0 (0.0) | 0 (0.0) |  |  |
| *T* allele | 140 (98.6) | 183 (98.4) |  | 1.000 |
| *G* allele | 2 (1.4) | 3 (1.6) |  |  |
| *fCD209 - 215* |  |  |  |  |
| *GG* | 54 (76.1) | 77 (82.8) |  | 0.351 |
| *GC* | 15 (21.1) | 12 (12.9) |  |  |
| *CC* | 2 (2.8) | 4 (4.3) |  |  |
| *G* allele | 123 (86.6) | 166 (89.3) |  | 0.494 |
| *C* allele | 19 (13.4) | 20 (10.8) |  |  |
| *fCD209 - 223* |  |  |  |  |
| *GG* | 50 (70.4) | 58 (62.4) |  | 0.577 |
| *GA* | 15 (21.1) | 24 (25.8) |  |  |
| *AA* | 6 (8.5) | 11 (11.8) |  |  |
| *G* allele | 115 (81.0) | 140 (75.3) |  | 0.231 |
| *A* allele | 27 (19.0) | 46 (24.7) |  |  |
| *fCD209 - 238* |  |  |  |  |
| *GG* | 65 (91.6) | 89 (95.7) |  | 0.481 |
| *GT* | 5 (7.0) | 3 (3.2) |  |  |
| *TT* | 1 (1.4) | 1 (1.0) |  |  |
| *G* allele | 135 (95.1) | 181 (97.3) |  | 0.376 |
| *T* allele | 7 (4.9) | 5 (2.7) |  |  |
| *fCD209 - 258* |  |  |  |  |
| *CC* | 63 (88.7) | 80 (86.0) |  | 0.857 |
| *CT* | 7 (9.9) | 10 (10.8) |  |  |
| *TT* | 1 (1.4) | 3 (3.2) |  |  |
| *C* allele | 133 (93.7) | 170 (91.4) |  | 0.531 |
| *T* allele | 9 (6.3) | 16 (8.6) |  |  |
| *fCD209 - 296* |  |  |  |  |
| *AA* | 63 (88.7) | 80 (86.0) |  | 0.669 |
| *AG* | 8 (11.3) | 11 (11.8) |  |  |
| *GG* | 0 (0.0) | 2 (2.2) |  |  |
| *A* allele | 134 (94.4) | 171 (91.9) |  | 0.514 |
| *G* allele | 8 (5.6) | 15 (8.1) |  |  |
| *fCD209 - 303* |  |  |  |  |
| *CC* | 44 (62.0) | 54 (58.1) |  | 0.458 |
| *CA* | 15 (21.1) | 27 (29.0) |  |  |
| *AA* | 12 (16.9) | 12 (12.9) |  |  |
| *C* allele | 103 (72.5) | 135 (72.6) |  | 1.000 |
| *A* allele | 39 (27.5) | 51 (27.4) |  |  |
| *fCD209 - 315* |  |  |  |  |
| *CC* | 26 (36.6) | 29 (31.2) |  | 0.625 |
| *CT* | 24 (33.8) | 38 (40.9) |  |  |
| *TT* | 21 (29.6) | 26 (28.0) |  |  |
| *C* allele | 76 (53.5) | 96 (51.6) |  | 0.739 |
| *T* allele | 66 (46.5) | 90 (48.4) |  |  |
| *fCD209 - 334* |  |  |  |  |
| *TT* | 63 (88.7) | 79 (85.0) |  | 0.421 |
| *TG* | 8 (11.3) | 11 (11.8) |  |  |
| *GG* | 0 (0.0) | 3 (3.2) |  |  |
| *T* allele | 134 (94.4) | 169 (90.9) |  | 0.296 |
| *G* allele | 8 (5.6) | 17 (9.1) |  |  |
| *fCD209 - 337* |  |  |  |  |
| *AA* | 56 (78.9) | 65 (69.9) |  | 0.416 |
| *AG* | 12 (16.9) | 23 (24.7) |  |  |
| *GG* | 3 (4.2) | 5 (5.4) |  |  |
| *A* allele | 124 (87.3) | 153 (82.3) |  | 0.223 |
| *G* allele | 18 (12.7) | 33 (17.7) |  |  |
| *fCD209 - 435* |  |  |  |  |
| *GG* | 66 (93.0) | 89 (95.7) |  | 0.580 |
| *GA* | 4 (5.6) | 4 (4.3) |  |  |
| *AA* | 1 (1.4) | 0 (0.0) |  |  |
| *G* allele | 136 (95.8) | 182 (97.9) |  | 0.339 |
| *A* allele | 6 (4.2) | 4 (2.2) |  |  |
| *fCD209 - 464* |  |  |  |  |
| *GG* | 65 (91.6) | 86 (92.5) |  | 1.000 |
| *GA* | 5 (7.0) | 6 (6.5) |  |  |
| *AA* | 1 (1.4) | 1 (1.1) |  |  |
| *G* allele | 135 (95.10 | 178 (95.70 |  | 0.796 |
| *A* allele | 7 (4.9) | 8 (4.3) |  |  |
| *fCD209 - 524* |  |  |  |  |
| *CC* | 63 (88.7) | 80 (86.0) |  | 0.669 |
| *CA* | 8 (11.3) | 11 (11.8) |  |  |
| *AA* | 0 (0.0) | 2 (2.2) |  |  |
| *C* allele | 134 (94.4) | 171 (91.9) |  | 0.514 |
| *A* allele | 8 (5.6) | 15 (8.1) |  |  |
| *fCD209 - 562* |  |  |  |  |
| *GG* | 66 (93.0) | 89 (95.7) |  | 0.580 |
| *GA* | 4 (5.6) | 4 (4.3) |  |  |
| *AA* | 1 (1.4) | 0 (0.0 |  |  |
| *G* allele | 136 (95.8) | 182 (97.9) |  | 0.339 |
| *A* allele | 6 (4.2) | 4 (2.2) |  |  |
| *fCD209 - 672* |  |  |  |  |
| *CC* | 44 (62.0) | 53 (57.0) |  | 0.602 |
| *CT* | 15 (21.1) | 26 (28.0) |  |  |
| *TT* | 12 (16.9) | 14 (15.1) |  |  |
| *C* allele | 103 (72.5) | 132 (71.0) |  | 0.805 |
| *T* allele | 39 (27.5) | 54 (29.0) |  |  |
| *fCD209 - 711* |  |  |  |  |
| *AA* | 26 (36.6) | 28 (30.1) |  | 0.562 |
| *AT* | 24 (33.8) | 39 (41.9) |  |  |
| *TT* | 21 (29.6) | 26 (28.0) |  |  |
| *A* allele | 76 (53.5) | 95 (51.1) |  | 0.738 |
| *T* allele | 66 (46.5) | 91 (48.9) |  |  |
| *fCD209 - 812* |  |  |  |  |
| *TT* | 48 (67.6) | 72 (77.4) |  | 0.325 |
| *TC* | 19 (26.8) | 26 (17.2) |  |  |
| *CC* | 4 (5.6) | 5 (5.4) |  |  |
| *T* allele | 115 (90.0) | 160 (86.0) |  | 0.229 |
| *C* allele | 27 (19.0) | 26 (14.0) |  |  |
| *fCD209 - 815* |  |  |  |  |
| *GG* | 49 (69.0) | 72 (77.4) |  | 0.327 |
| *GA* | 19 (26.8) | 16 (17.2) |  |  |
| *AA* | 3 (4.2) | 5 (5.4) |  |  |
| *G* allele | 117 (82.4) | 160 (86.0) |  | 0.442 |
| *A* allele | 25 (17.6) | 26 (14.0) |  |  |
| *fCD209 - 872* |  |  |  |  |
| *AA* | 49 (69.0) | 73 (78.5) |  | 0.269 |
| *AC* | 19 (26.8) | 15 (16.1) |  |  |
| *CC* | 3 (4.2) | 5 (5.4) |  |  |
| *A* allele | 117 (82.4) | 161 (86.6) |  | 0.353 |
| *C* allele | 25 (17.6) | 25 (13.4) |  |  |
| *fCD209 - 880* |  |  |  |  |
| *AA* | 49 (69.0) | 73 (78.5) |  | 0.318 |
| *AT* | 18 (25.4) | 15 (16.1) |  |  |
| *TT* | 4 (5.6) | 5 (5.4) |  |  |
| *A* allele | 116 (81.7) | 161 (86.6) |  | 0.282 |
| *T* allele | 26 (18.3) | 25 (13.4) |  |  |
| *fCD209 - 883* |  |  |  |  |
| *GG* | 49 (69.0) | 72 (77.4) |  | 0.327 |
| *GT* | 19 (26.8) | 16 (17.2) |  |  |
| *TT* | 3 (4.2) | 5 (5.4) |  |  |
| *G* allele | 117 (82.4) | 160 (86.0) |  | 0.442 |
| *T* allele | 25 (17.6) | 26 (14.0) |  |  |
| *fCD209 - 910* |  |  |  |  |
| *GG* | 47 (66.2) | 56 (60.2) |  | 0.524 |
| *GA* | 21 (29.6) | 29 (31.2) |  |  |
| *AA* | 3 (4.2) | 8 (8.6) |  |  |
| *G* allele | 115 (81.0) | 141 (75.8) |  | 0.284 |
| *A* allele | 27 (19.0) | 45 (24.2) |  |  |
| *fCD209 - 943* |  |  |  |  |
| *GG* | 64 (90.1) | 81 (87.1) |  | 0.628 |
| *GA* | 7 (9.9) | 12 (12.9) |  |  |
| *AA* | 0 (0.0) | 0 (0.0) |  |  |
| *G* allele | 135 (95.1) | 174 (93.6) |  | 0.639 |
| *A* allele | 7 (4.9) | 12 (6.5) |  |  |
| *fCD209 - 994* |  |  |  |  |
| *TT* | 49 (69.0) | 73 (78.5) |  | 0.146 |
| *TC* | 20 (28.2) | 15 (16.1) |  |  |
| *CC* | 2 (2.8) | 5 (5.4) |  |  |
| *T* allele | 118 (83.1) | 161 (86.6) |  | 0.435 |
| *C* allele | 24 (16.9) | 25 (13.4) |  |  |
| *fCD209 + 1900* |  |  |  |  |
| *GG* | 58 (81.7) | 88 (94.6) | 3.95 (1.3 - 11.7) | 0.011 |
| *GA* | 13 (18.3) | 5 (5.4) |  |  |
| *AA* | 0 (0.0) | 0 (0.0) |  |  |
| *G* allele | 129 (90.9) | 181 (97.3) | 3.65 (1.3 - 10.5) | 0.014 |
| *A* allele | 13 (9.2) | 5 (2.7) |  |  |
| *fCD209 + 1952* |  |  |  |  |
| *CC* | 71 (100.0) | 91 (97.9) |  | 0.506 |
| *CA* | 0 (0.0) | 2 (2.2) |  |  |
| *AA* | 0 (0.0) | 0 (0.0) |  |  |
| *C* allele | 0 (0.0) | 2 (1.08) |  | 0.508 |
| *A* allele | 142 (100.0) | 184 (98.9) |  |  |
| *fCD209 + 2076* |  |  |  |  |
| *CC* | 58 (81.7) | 74 (79.6) |  | 0.333 |
| *CT* | 6 (8.5) | 4 (4.3) |  |  |
| *TT* | 7 (9.9) | 15 (16.1) |  |  |
| *C* allele | 122 (85.9) | 152 (81.7) |  | 0.368 |
| *T* allele | 20 (14.1) | 34 (18.3) |  |  |
| *fCD209 + 2126* |  |  |  |  |
| *TT* | 51 (71.8) | 69 (74.2) |  | 0.745 |
| *TC* | 7 (9.9) | 6 (6.5) |  |  |
| *CC* | 13 (18.3) | 18 (19.4) |  |  |
| *T* allele | 109 (76.8) | 144 (77.4) |  | 0.895 |
| *C* allele | 33 (23.2) | 42 (22.6) |  |  |
| *fCD209 + 2127* |  |  |  |  |
| *GG* | 71 (100.0) | 90 (96.8) |  | 0.506 |
| *GA* | 0 (0.0) | 2 (2.2) |  |  |
| *AA* | 0 (0.0) | 1 (1.08) |  |  |
| *G* allele | 142 (100.0) | 182 (97.9) |  | 0.136 |
| *A* allele | 0 (0.0) | 4 (2.2) |  |  |
| *fCD209 + 2217* |  |  |  |  |
| *CC* | 62 (87.3) | 83 (89.2) |  | 0.752 |
| *CG* | 6 (8.5) | 5 (5.4) |  |  |
| *GG* | 3 (4.2) | 5 (5.4) |  |  |
| *C* allele | 130 (91.5) | 171 (91.9) |  | 1.000 |
| *G* allele | 12 (8.5) | 15 (8.1) |  |  |
| *fCD209 + 2276* |  |  |  |  |
| *CC* | 71 (100.0) | 88 (94.6) |  | 0.134 |
| *CT* | 0 (0.0) | 4 (4.3) |  |  |
| *TT* | 0 (0.0) | 1 (1.1) |  |  |
| *C* allele | 142 (100.0) | 180 (96.8) | NA^b^ | 0.038 |
| *T* allele | 0 (0.0) | 6 (3.2) |  |  |
| *fCD209 + 2286* |  |  |  |  |
| *GG* | 37 (52.1) | 47 (50.5) |  | 0.856 |
| *GA* | 2 (2.8) | 5 (5.4) |  |  |
| *AA* | 32 (45.1) | 41 (44.1) |  |  |
| *G* allele | 76 (53.5) | 99 (53.2) |  | 1.000 |
| *A* allele | 66 (46.5) | 87 (46.8) |  |  |
| *fCD209 + 2334* |  |  |  |  |
| *CC* | 61 (85.9) | 87 (93.5) |  | 0.221 |
| *CT* | 8 (11.3) | 4 (4.3) |  |  |
| *TT* | 2 (2.8) | 2 (2.2) |  |  |
| *C* allele | 130 (91.5) | 178 (95.7) |  | 0.162 |
| *T* allele | 12 (8.5) | 8 (4.3) |  |  |
| *fCD209 + 2336* |  |  |  |  |
| *CC* | 39 (54.9) | 47 (50.5) |  | 0.710 |
| *CT* | 2 (2.8) | 5 (5.4) |  |  |
| *TT* | 30 (42.3) | 41 (44.1) |  |  |
| *C* allele | 80 (56.3) | 99 (53.2) |  | 0.578 |
| *T* allele | 62 (43.7) | 87 (46.8) |  |  |
| *fCD209 + 2338* |  |  |  |  |
| *CC* | 38 (53.5) | 47 (50.5) |  | 0.796 |
| *CT* | 2 (2.8) | 5 (5.4) |  |  |
| *TT* | 31 (43.7) | 41 (44.1) |  |  |
| *C* allele | 78 (54.9) | 99 (53.2) |  | 0.823 |
| *T* allele | 64 (45.1) | 87 (46.8) |  |  |
| *fCD209 + 2388* |  |  |  |  |
| *TT* | 63 (88.7) | 90 (96.8) |  | 0.095 |
| *TC* | 6 (8.5) | 2 (2.2) |  |  |
| *CC* | 2 (2.8) | 1 (1.1) |  |  |
| *T* allele | 132 (93.0) | 182 (97.8) |  | 0.050 |
| *C* allele | 10 (7.0) | 4 (2.20 |  |  |
| *fCD209 + 2391* |  |  |  |  |
| *CC* | 65 (91.5) | 90 (94.6) |  | 0.413 |
| *CT* | 0 (0.0) | 1 (1.1) |  |  |
| *TT* | 6 (8.5) | 4 (4.3) |  |  |
| *C* allele | 130 (91.5) | 177 (95.2) |  | 0.255 |
| *T* allele | 12 (8.5) | 9 (4.8) |  |  |
| *fCD209 + 2392* |  |  |  |  |
| *GG* | 66 (93.0) | 76 (81.7) |  | 0.057 |
| *GA* | 0 (0.0) | 4 (4.3) |  |  |
| *AA* | 5 (7.0) | 13 (14.0) |  |  |
| *G* allele | 132 (93.0) | 156 (83.9) | 2.57 (1.2 - 5.5) | 0.016 |
| *A* allele | 10 (7.0) | 30 (16.1) |  |  |
| *fCD209 + 2440* |  |  |  |  |
| *GG* | 65 (91.5) | 88 (94.6) |  | 0.414 |
| *GA* | 0 (0.0) | 1 (1.1) |  |  |
| *AA* | 6 (8.5) | 4 (4.3) |  |  |
| *G* allele | 130 (91.5) | 177 (95.2) |  | 0.255 |
| *A* allele | 12 (8.5) | 9 (4.8) |  |  |
| *fCD209 + 2457* |  |  |  |  |
| *CC* | 37 (52.1) | 44 (47.3) |  | 0L465 |
| *CT* | 2 (2.8) | 7 (7.5) |  |  |
| *TT* | 32 (45.1) | 42 (45.2) |  |  |
| *C* allele | 76 (53.5) | 95 (51.1) |  | 0.738 |
| *T* allele | 66 (46.5) | 91 (48.9) |  |  |
| *fCD209 + 2469* |  |  |  |  |
| *CC* | 67 (94.4) | 84 (90.3) |  | 0.676 |
| *CT* | 2 (2.8) | 3 (3.2) |  |  |
| *TT* | 2 (2.8) | 6 (6.5) |  |  |
| *C* allele | 136 (95.8) | 171 (91.9) |  | 0.179 |
| *T* allele | 6 (4.2) | 15 (8.1) |  |  |
| *fCD209 + 2498* |  |  |  |  |
| *GG* | 65 (91.5) | 88 (94.6) |  | 0.414 |
| *GT* | 0 (0.0) | 1 (1.1) |  |  |
| *TT* | 6 (8.5) | 4 (4.3) |  |  |
| *G* allele | 130 (91.5) | 177 (95.2) |  | 0.255 |
| *T* allele | 12 (8.5) | 9 (4.8) |  |  |
| *fCD209 + 2598* |  |  |  |  |
| *GG* | 39 (54.9) | 47 (50.5) |  | 0.440 |
| *GA* | 1 (1.4) | 5 (5.4) |  |  |
| *AA* | 31 (43.7) | 41 (44.1) |  |  |
| *G* allele | 79 (55.6) | 99 (53.2) |  | 0.737 |
| *A* allele | 63 (44.4) | 87 (46.8) |  |  |
| *fCD209 + 2713* |  |  |  |  |
| *CC* | 46 (64.8) | 71 (76.3) |  | 0.225 |
| *CT* | 6 (8.5) | 6 (6.5) |  |  |
| *TT* | 19 (26.8) | 16 (17.2) |  |  |
| *C* allele | 98 (69.0) | 148 (79.6) | 1.75 (1.1 - 2.9) | 0.039 |
| *T* allele | 44 (31.0) | 38 (20.4) |  |  |
| *fCD209 + 2718* |  |  |  |  |
| *CC* | 66 (93.0) | 84 (90.3) |  | 0.112 |
| *CT* | 5 (7.0) | 4 (4.3) |  |  |
| *TT* | 0 (0.0) | 5 (5.4) |  |  |
| *C* allele | 137 (96.5) | 172 (92.5) |  | 0.155 |
| *T* allele | 5 (3.5) | 14 (7.5) |  |  |
| *fCD209 + 2723* |  |  |  |  |
| *TT* | 64 (90.1) | 82 (88.2) |  | 0.435 |
| *GT* | 6 (8.5) | 11 (11.8) |  |  |
| *GG* | 1 (1.4) | 0 (0.0) |  |  |
| *T* allele | 134 (94.4) | 175 (94.1) |  | 1.000 |
| *G* allele | 8 (5.6) | 11 (5.9) |  |  |
| *fCD209 + 2797* |  |  |  |  |
| *TT* | 45 (63.4) | 60 (64.5) |  | 1.000 |
| *TG* | 6 (8.5) | 7 (7.5) |  |  |
| *GG* | 20 (28.2) | 26 (28.0) |  |  |
| *T* allele | 96 (67.6) | 127 (68.3) |  | 0.905 |
| *G* allele | 46 (32.4) | 59 (31.7) |  |  |
| *fCD209 + 2871* |  |  |  |  |
| *GG* | 46 (64.8) | 68 (73.1) |  | 0.475 |
| *GC* | 10 (14.1) | 8 (8.6) |  |  |
| *CC* | 15 (21.1) | 17 (18.3) |  |  |
| *G* allele | 102 (71.8) | 144 (77.4) |  | 0.437 |
| *C* allele | 40 (28.2) | 42 (22.6) |  |  |
| *fCD209 + 3070* |  |  |  |  |
| *TT* | 67 (94.4) | 85 (91.4) |  | 0.274 |
| *TC* | 1 (1.4) | 0 (0.0) |  |  |
| *CC* | 3 (4.2) | 8 (8.6) |  |  |
| *T* allele | 135 (95.1) | 170 (91.4) |  | 0.275 |
| *C* allele | 7 (4.9) | 16 (8.6) |  |  |

^a^ Odds Ratio.

^b^ Not available.
