# Supplementary material for: Polymorphisms in the feline TNFA and CD209 genes are associated with the outcome of feline coronavirus infection
Source: Vet Res. 2014 Dec 16;45(1):123. doi: 10.1186/s13567-014-0123-6 (PMC4267428; doi:10.1186/s13567-014-0123-6)
Supplement: Additional file 3: — Frequencies of the genotypes and alleles of the proposed FIP-associated SNPs in Birman cats and associations with FIP. Neither the percentage of genotypes nor alleles was found to be associated with the outcome of FCoV infection in disease and non-disease group. [file 13567_2014_123_MOESM3_ESM.doc]

**Additional file 3 Frequencies of the genotypes and alleles of the proposed FIP-associated SNPs in Birman cats and associations with FIP.**

| SNP | FIP  number (%) | Non FIP number (%) | *P* value |
| --- | --- | --- | --- |
| *A2.191286425* |  |  |  |
| *CC* | 56 (78.9) | 80 (86.0) | 0.200 |
| *CT* | 13 (18.3) | 13 (14.0) |  |
| *TT* | 2 (2.8) | 0 (0.0) |  |
| *C* allele | 125 (88.0) | 173 (93.0) | 0.127 |
| *T* allele | 17 (12.0) | 13 (7.0) |  |
| *A1.196617776* |  |  |  |
| *CC* | 25 (35.2) | 31 (33.3) | 0.960 |
| *CA* | 27 (38.0) | 36 (38.7) |  |
| *AA* | 19 (26.8) | 26 (28.0) |  |
| *C* allele | 77 (54.2) | 98 (52.7) | 0.824 |
| *A* allele | 65 (45.8) | 88 (47.3) |  |
| *A1.206840008* |  |  |  |
| *GG* | 30 (42.3) | 33 (35.5) | 0.203 |
| *GA* | 24 (33.8) | 44 (47.3) |  |
| *AA* | 17 (23.9) | 16 (17.2) |  |
| *G* allele | 84 (59.2) | 110 (59.1) | 1.000 |
| *A* allele | 58 (40.9) | 76 (40.9) |  |
| *Un.59861682* |  |  |  |
| *GG* | 39 (54.9) | 52 (55.9) | 0.243 |
| *GA* | 26 (36.6) | 26 (28.0) |  |
| *AA* | 6 (8.5) | 15 (16.1) |  |
| *G* allele | 104 (73.2) | 130 (69.9) | 0.539 |
| *A* allele | 38 (26.8) | 56 (30.1) |  |
| *E2.65509996* |  |  |  |
| *CC* | 60 (84.5) | 77 (82.8) | 0.872 |
| *CT* | 10 (14.1) | 13 (14.0) |  |
| *TT* | 1 (1.4) | 3 (3.2) |  |
| *C* allele | 130 (91.6) | 167 (89.8) | 0.704 |
| *T* allele | 12 (8.5) | 19 (10.2) |  |
